# G Tolerance Prediction Model Using Mobile Device–Measured Cardiac Force Index for Military Aircrew: Observational Study

**DOI:** 10.2196/48812

**Published:** 2023-07-26

**Authors:** Ming-Hao Kuo, You-Jin Lin, Wun-Wei Huang, Kwo-Tsao Chiang, Min-Yu Tu, Chi-Ming Chu, Chung-Yu Lai

**Affiliations:** 1 Graduate Institute of Medical Sciences National Defense Medical Center Taipei City Taiwan; 2 Thoracic Department China Medical University Beigang Hospital Yunlin County Taiwan; 3 Aviation Physiology Research Laboratory Kaohsiung Armed Forces General Hospital Gangshan Branch Kaohsiung City Taiwan; 4 Superintendent Office Taipei Veterans General Hospital Fonglin Branch Hualien County Taiwan; 5 School of Public Health National Defense Medical Center Taipei City Taiwan; 6 Graduate Institute of Aerospace and Undersea Medicine National Defense Medical Center Taipei City Taiwan; 7 Orthopedics Division Taichung Armed Forces General Hospital Taichung City Taiwan; 8 Department of Health Business Administration Meiho University Pingtung County Taiwan; 9 Department of Life Sciences National Chung Hsing University Taichung City Taiwan; 10 Graduate Institute of Life Sciences National Defense Medical Center Taipei City Taiwan; 11 Big Data Research Center College of Medicine Fu-Jen Catholic University New Taipei City Taiwan; 12 Department of Public Health Kaohsiung Medical University Kaohsiung City Taiwan; 13 Department of Public Health China Medical University Taichung City Taiwan

**Keywords:** G force, baroreflex, anti-G straining maneuver, G tolerance, cardiac force index, anti-G suit, relaxed G tolerance, straining G tolerance, cardiac force ratio

## Abstract

**Background:**

During flight, G force compels blood to stay in leg muscles and reduces blood flow to the heart. Cardiovascular responses activated by the autonomic nerve system and strengthened by anti-G straining maneuvers can alleviate the challenges faced during G loading. To our knowledge, no definite cardiac information measured using a mobile health device exists for analyzing G tolerance. However, our previous study developed the cardiac force index (CFI) for analyzing the G tolerance of military aircrew.

**Objective:**

This study used the CFI to verify participants’ cardiac performance when walking and obtained a formula for predicting an individual’s G tolerance during centrifuge training.

**Methods:**

Participants from an air force aircrew undertook high-G training from January 2020 to December 2022. Their heart rate (HR) in beats per minute and activity level per second were recorded using the wearable BioHarness 3.0 device. The CFI was computed using the following formula: *weight × activity / HR during resting or walking*. Relaxed G tolerance (RGT) and straining G tolerance (SGT) were assessed at a slowly increasing rate of G loading (0.1 G/s) during training. Other demographic factors were included in the multivariate regression to generate a model for predicting G tolerance from the CFI.

**Results:**

A total of 213 eligible trainees from a military aircrew were recruited. The average age was 25.61 (SD 3.66) years, and 13.1% (28/213) of the participants were women. The mean resting CFI and walking CFI (WCFI) were 0.016 (SD 0.001) and 0.141 (SD 0.037) kg × G/beats per minute, respectively. The models for predicting RGT and SGT were as follows: *RGT = 0.066 × age + 0.043 × (WCFI × 100) – 0.037 × height + 0.015 × systolic blood pressure – 0.010 × HR + 7.724* and *SGT = 0.103 × (WCFI × 100) − 0.069 × height + 0.018 × systolic blood pressure + 15.899*. Thus, the WCFI is a positive factor for predicting the RGT and SGT before centrifuge training.

**Conclusions:**

The WCFI is a vital component of the formula for estimating G tolerance prior to training. The WCFI can be used to monitor physiological conditions against G stress.

## Introduction

### Background

On the Earth’s surface, humans are exposed to gravitational forces. The applied acceleration of gravity, 9.8 m/s^2^, is defined as 1 Gz (1 G) in the direction from the head to the feet when a person is standing vertically. During flight, changes in speed or direction result in acceleration. The same magnitude of inertial force is generated in the opposite direction of the acceleration. Because fighter aircrafts are highly agile, military pilots experience high levels of G force, which decrease blood pressure and cause massive shifts in and redistributions of bodily fluid, especially during acrobatic combat maneuvers. The cardiovascular system is highly sensitive to G force, and its ability to maintain sufficient cerebral perfusion can be impaired under high-G force. Military pilots commonly experience visual degradation (eg, grayouts and blackouts) due to a decrease in the blood volume entering the retina [1-3]. If the supply of blood to the brain ceases, a military pilot experiences G-induced loss of consciousness (GLOC). In such cases, the pilot loses their ability to manipulate the aircraft, and a catastrophic event may occur.

Baroreflex is a well-known compensatory regulation activated by decreases in the arterial baroreceptor input under G load. The physiological reactions that are usually observed are an elevated heart rate (HR), increased peripheral vessel resistance, and greater cardiac contractility moderated by the autonomic nerve system [4,5]. The anti-G straining maneuver (AGSM) is considered the most crucial technique for increasing the cardiovascular system’s ability to withstand G stress [6-8]. Additionally, several studies have found that anthropometric parameters are associated with G tolerance [9-11].

Because no appropriate integrated cardiac parameter exists for monitoring G tolerance, we successfully introduced the cardiac force index (CFI) into the high-G training undergone by military aircrew [12]. Initially, the CFI was monitored using a wearable device and used to predict the running performance of military academy students [13-16]. The CFI consists of 3 factors that are relevant to G tolerance, namely body weight, dynamic changes in acceleration, and HR. The findings of the aforementioned studies revealed that the walking CFI (WCFI)—that is, the CFI while an individual is walking on the ground—was significantly positively correlated with G tolerance, as determined through centrifuge training.

### Objective

High-G training is commonly used to assess the G tolerance of military pilots and determine whether they are fit to fly a modern jet. To our best knowledge, almost no studies have designed a model for predicting the G tolerance of aircrew before the training. Our previous study demonstrated that the WCFI calculated using mobile health technology can be used to identify potential factors affecting the ability to withstand G levels.

Followed the former finding, we attempted to develop a mathematical formula for predicting G tolerance on the basis of the CFI, which can be calculated before the beginning of training. Therefore, we can measure cardiac health and detect the low G tolerance of military pilots via mobile wearable devices during daily activity. In the future, we will try to establish the strategy of rapid G-resistance ability assessment by monitoring mobile cardiac data before the flight and to ensure pilots’ safety during flight missions.

## Methods

### Study Design and Participants

This longitudinal, observational study was conducted to evaluate the relationship between the CFI and G tolerance. The acceleration rate was set to 0.1 G/s during training. We also developed a formula for using CFI data to determine the level of G tolerance.

The participants were air force aircrew trainees attending high-G training at the Aviation Physiology Research Laboratory (APRL), Kaohsiung City, Taiwan. The participants were required to undergo medical examinations and meet the standards to be deemed fit for centrifuge training, which was conducted from January 2020 to December 2022.

### Ethics Approval and Informed Consent

The documents and permission to perform this study were issued by the ethics committee of the Institutional Review Board of Kaohsiung Armed Forces General Hospital in Taiwan (approvals KAFGH 109-001 and 110-009). Before the study, written informed consent was provided by each participant to ensure that they understood the purpose and content of the study.

### Protocol of Cardiac Data Collection

**Figure 1:**
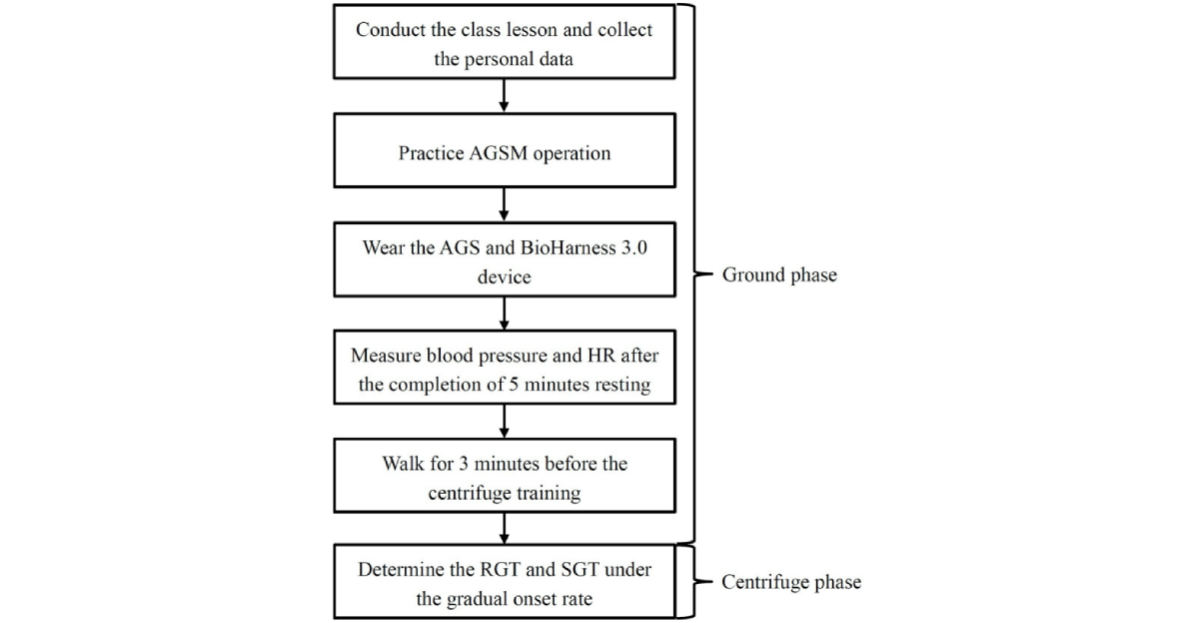
Study protocol. AGS: anti-G suit; AGSM: anti-G straining maneuver; HR: heart rate; RGT: relaxed G tolerance; SGT: straining G tolerance.

Air force aircrew attended a 1-day high-G training at the APRL. Their cardiovascular performance at rest and while walking was monitored using mobile technology and sensors. Centrifuge training was performed to simulate the hypergravitational environment and examine their G tolerance. A flowchart of the study protocol is presented in Figure 1.

#### Mobile Monitoring Device

This study used a mobile health (mHealth) BioHarness 3.0 module (Zephyr Technology Corporation), which is noninvasive and equipped with a gyroscope and accelerometers.

Activity and HR were the 2 key indicators in this study. The BioHarness 3.0 sensor detected the distance that the participants moved by using its internal 3-axis accelerometers and calculated the activity per second. Activity levels were recorded using piezoelectric technology and are presented as the square roots of the acceleration values in the x, y, and z directions.

HR is presented as the number of beats per minute (bpm) and was measured using a conductive electrode sensor, with the thoracic loop strap fitted elastically to the skin over the thorax. To reduce noise during body movement, a shoulder strap was used to minimize the displacement of the BioHarness 3.0 sensor [17].

#### Ground Phase

**Figure 2:**
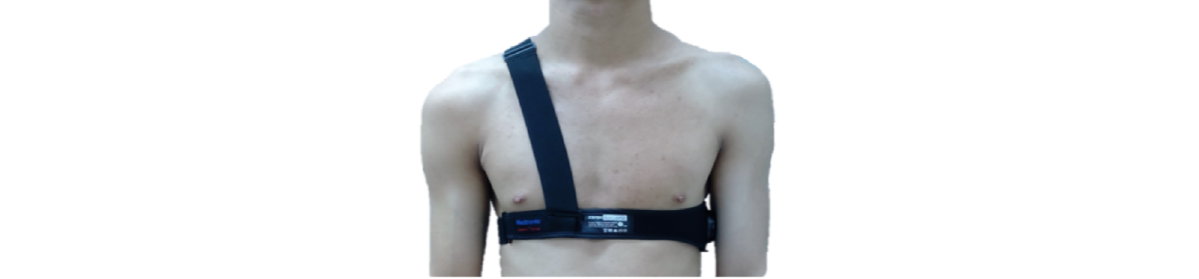
BioHarness 3.0 module with chest and shoulder straps.

**Figure 3:**
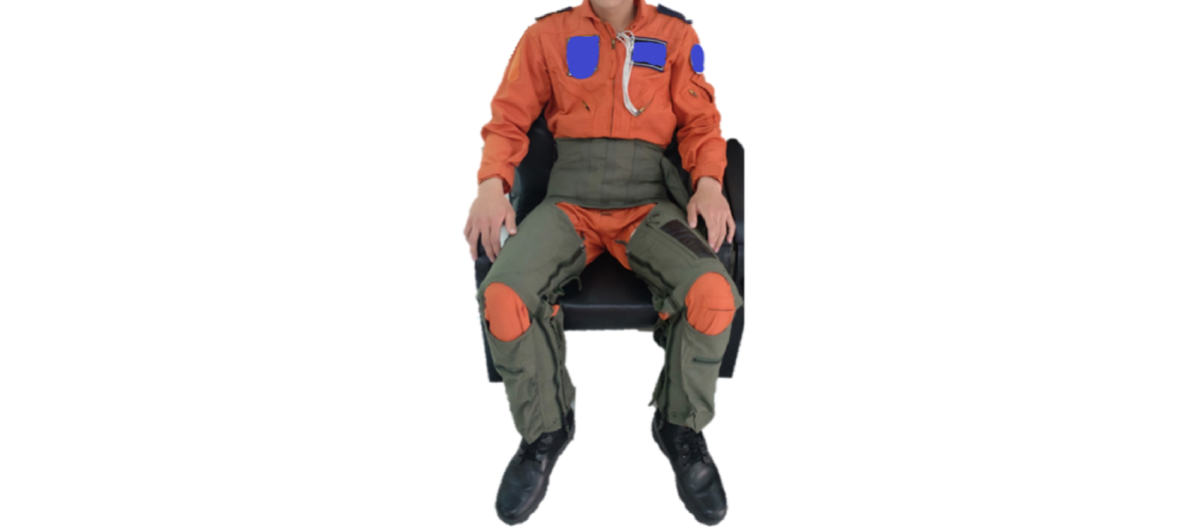
Resting for 5 minutes with an uninflated anti-G suit.

The instructor, who was an aviation physiology officer at the APRL, held a lecture on acceleration physiology and G awareness. After the lesson, we explained the study protocol to aircrew willing to participate and used a questionnaire to collect their personal data, namely their birth year, gender, height, and weight; whether they smoked; whether they drank alcohol; and their exercise habits. Thereafter, all aircrew mastered the AGSM, and the instructor examined the participants’ execution of the AGSM before G-tolerance tests were performed. The 2 main components of the AGSM are the holding of a preparatory breath against the closed glottis every 3 seconds followed by rapid air exchange and isometric muscle tensing with an emphasis on the legs, buttocks, and abdomen. The trainees wore a standard anti-G suit (AGS) and were outfitted with a BioHarness 3.0 sensor, which was placed under the left central armpit and strapped to the chest and shoulder (Figure 2). After the fit of the AGS was checked, we pressed the central button to power on the BioHarness 3.0 sensor and started collecting cardiac data. After the participants had rested for 5 minutes in a chair, their systolic blood pressure (SBP), diastolic blood pressure (DBP), and HR were evaluated using an Omron 1100U sphygmomanometer (Omron Healthcare Company; Figure 3).

After the resting data had been obtained, the participants performed relaxed and normal walking for 3 minutes. The participants performed squats before and after walking. Walking data could be obtained from the changes in activity identified by the 3-axis accelerometers.

#### Centrifuge Phase

After they had completed the ground phase, the trainees entered the human centrifuge gondola (Latécoère) at the APRL. The human centrifuge trained 1 person at a time, and the maximum training capacity was 8 trainees per day. The length of the centrifuge’s arm was 20 feet (6.1 meters). The hydraulic power system could achieve a training onset rate and G level of up to 6 G/s and 9 G, respectively.

The participant wore a safety belt and sat on the simulated cockpit seat, which leaned back by 13°. After the foot pedals had been adjusted, the participant practiced the AGSM again and then rested for 2 minutes in a seated position. The APRL instructor started the centrifuge at an idle run of 1.4 G. Before the trainee’s G tolerance was assessed, the instructor accelerated the centrifuge at an onset rate of 0.1 G/s. The participant’s relaxed G tolerance (RGT) and straining G tolerance (SGT) were determined without inflating the AGS [11]. The RGT value was defined as the G value at which the participant experienced complete loss of peripheral vision or 50% loss of their central vision in the relaxed state. Thereafter, the participant commenced the AGSM to resist the physiological effect of G force as the level of centrifugation was increased. The SGT value was the G value at which the participant experienced the same visual degradation or a G force equal to the upper limit of 9 G. The level of visual loss for each participant was analyzed using the light bar in front of them inside the gondola [11].

#### Data Handling and Conversion Procedure

The mHealth BioHarness 3.0 device obtained data every second on the participants while they were on the ground. We used the charging and configuration cradle to download and save the digital data to a folder named “Summary.” If the signal values of the HR confidence or system confidence were below 20%, the data were considered unstable and unreliable [18]. There were 219 military aircrew members enrolled in this study. However, 6 participants were excluded from the analysis due to poor data quality, resulting in a final sample size of 213 participants. Resting and walking data were extracted and analyzed using previously proposed research methods [12,13].

Regarding the digital data, the activity and HR variables combined with the individual’s body weight were used to calculate the CFI and cardiac force ratio (CFR). For every second for which data were collected, the weight and activity values were multiplied and divided by the HR. The mathematical formula was as follows: *CFI = weight × activity / HR* [13,14]. The average resting CFI (RCFI) and WCFI on the ground over a 2-minute period were calculated, and the CFR was obtained by dividing the WCFI by the RCFI.

### Statistical Analysis

Descriptive statistics were calculated, and continuous variables are presented as means, SDs, and ranges. We used values and proportions to describe discrete data.

In the statistical analysis, the relationship between cardiac function on the ground and G tolerance in the centrifuge was assessed using Pearson correlation. A model for predicting G tolerance that is connected to the CFI was developed using stepwise multiple linear regression and by adjusting other covariates.

Statistical analyses were conducted using SPSS software (version 27.0; IBM Corp). Two-tailed *P* values <.05 were considered significant.

## Results

### Analysis of Demographic Data

The demographic data are displayed in Table 1. This study recruited 213 aircrew who finished the study. The average age of the participants was 25.61 (SD 3.66) years, and 13.1% (28/213) of the participants were women. The average height of the participants was 173.18 (SD 6.75) cm, their average weight was 70.39 (SD 11.44) kg, and their average BMI was 23.38 (SD 2.91) kg/m^2^. A total of 50 (23.5%) aircrew members smoked, 38 (17.8%) drank alcohol, and over half (n=114, 53.5%) habitually exercised.

**Table 1:** Characteristics of the enrolled aircrew (n=213).

Characteristic			Value
Age (years), mean (SD; range)			25.61 (3.66; 22-27)
Gender, women, n (%)			28 (13.1)
Height (cm), mean (SD; range)			173.18 (6.75; 156-188)
Weight (kg), mean (SD; range)			70.39 (11.44; 48-99)
BMI (kg/m^2^), mean (SD; range)			23.38 (2.91; 17.31-32.70)
**Smoking status, n (%)**			
	No	163 (76.5)	
	Yes	50 (23.5)	
**Drinking status, n (%)**			
	No	175 (82.2)	
	Yes	38 (17.8)	
**Habitually exercised, n (%)**			
	No	99 (46.5)	
	Yes	114 (53.5)	

### Physiological Recordings on the Ground or Before Centrifuge Training

The changes in cardiovascular responses are listed in Table 2. The mean SBP, DBP, and HR while sitting and before centrifuge training were 140.40 (SD 14.47) mm Hg, 81.42 (SD 8.33) mm Hg, and 88.56 (SD 15.33) bpm, respectively. The mean WCFI was much higher than the mean RCFI (WCFI: 0.141, SD 0.037 vs RCFI: 0.016, SD 0.001 kg × G/bpm). The average CFR, computed by dividing the WCFI by the RCFI, was 10.76 (SD 4.38). During the G tolerance test, the RGT and SGT were 4.9 (SD 0.9) and 7.9 (SD 1.1) G, respectively, under a slow onset rate. Out of 213 aircrew members, 23 (10.9%) had a RGT greater than 6 G, and 60 (28.2%) had an SGT greater than 8 G (Tables 3 and 4). Pearson correlation coefficients were used to determine the relationship of RGT with SBP (*r*=.149; *P*=.03), HR (*r*=−.187; *P*=.006), and WCFI (*r*=.234; *P*=.001). Additionally, SGT was positively associated with SBP (*r*=.167; *P*=.02), DBP (*r*=.199; *P*=.01), and WCFI (*r*=.256; *P*<.001), as shown in Table 5.

**Table 2:** Descriptive analysis of cardiovascular and physiological information.

Variables	Value, mean (SD; range)
SBP^a^ (mm Hg)	140.40 (14.47; 102-177)
DBP^b^ (mm Hg)	81.42 (8.33; 50-107)
HR^c^ (bpm^d^)	88.56 (15.33; 56-145)
RCFI^e^ (kg × G/bpm)	0.016 (0.001; 0.006-0.088)
WCFI^f^ (kg × G/bpm)	0.141 (0.037; 0.020-0.266)
CFR^g^	10.76 (4.38; 1.52-23.02)

^a^SBP: systolic blood pressure.

^b^DBP: diastolic blood pressure.

^c^HR: heart rate.

^d^bpm: beats per minute.

^e^RCFI: resting cardiac force index.

^f^WCFI: walking cardiac force index.

^g^CFR: cardiac force ratio.

**Table 3:** Relaxed G tolerance distribution.

Relaxed G tolerance (G)	Participant (n=213), n (%)
3.0-3.4	8 (3.8)
3.5-3.9	17 (8)
4.0-4.4	40 (18.8)
4.5-4.9	53 (24.9)
5.0-5.4	45 (21.1)
5.5-5.9	27 (12.7)
6.0-6.4	10 (4.7)
6.5-6.9	8 (3.8)
7.0-7.4	3 (1.4)
7.5-7.9	1 (0.5)
8.0-8.4	1 (0.5)

**Table 4:** Straining G tolerance distribution.

Straining G tolerance (G)	Participant (n=213), n (%)
4.5-4.9	1 (0.5)
5.0-5.4	4 (1.9)
5.5-5.9	9 (4.2)
6.0-6.4	13 (6.1)
6.5-6.9	18 (8.5)
7.0-7.4	19 (8.9)
7.5-7.9	36 (16.9)
8.0-8.4	35 (16.4)
8.5-8.9	18 (8.5)
9.0	60 (28.2)

**Table 5:** Pearson correlation coefficients between G tolerance and cardiac function.

Variables			RGT^a^		SGT^b^		SBP^c^		DBP^d^		HR^e^		RCFI^f^		WCFI^g^		CFR^h^
**RGT**																	
	*r*	1		.535		.149		.127		–.187		.087		.234		–.025	
	*P* value	—		*<*.001		.03		.07		.006		.20		.001		.72	
**SGT**																	
	*r*	.535		1		.167		.199		–.111		.124		.256		–.048	
	*P* value	*<*.001		—		.02		.01		.11		.07		*<*.001		.49	
**SBP**																	
	*r*	.149		.167		1		.519		.181		–.034		.245		.068	
	*P* value	.03		.02		—		*<*.001		.008		.62		*<*.001		.07	
**DBP**																	
	*r*	.127		.199		.519		1		.372		–.020		.091		.060	
	*P* value	.07		.01		*<*.001		—		*<*.001		.78		.19		.38	
**HR**																	
	*r*	–.187		–.111		.181		.372		1		–.310		–.337		.203	
	*P* value	.006		.11		.008		*<*.001		—		*<*.001		*<*.001		.003	
**RCFI**																	
	*r*	.087		.124		–.034		–.020		–.310		1		.329		–.724	
	*P* value	.20		.07		.62		.78		*<*.001		—		*<*.001		*<*.001	
**WCFI**																	
	*r*	.234		.256		.245		.091		–.337		.329		1		.177	
	*P* value	.001		*<*.001		*<*.001		.19		*<*.001		*<*.001		—		.009	
**CFR**																	
	*r*	–.025		–.048		.068		.060		.203		–.724		.177		1	
	*P* value	.72		.49		.07		.38		.003		*<*.001		.009		—	

^a^RGT: relaxed G tolerance.

^b^SGT: straining G tolerance.

^c^SBP: systolic blood pressure.

^d^DBP: diastolic blood pressure.

^e^HR: heart rate.

^f^RCFI: resting cardiac force index.

^g^WCFI: walking cardiac force index.

^h^CFR: cardiac force ratio.

### Model for Predicting G Tolerance From the CFI Through Multivariate Linear Regression

As shown in Table 6, the model for predicting G tolerance was established using multivariate linear regression with stepwise selection. The WCFI was found to be the significant parameter for predicting RGT (*P*=.01) and SGT (*P*<.001). The formula for predicting RGT was as follows: *RGT = 0.066 × age + 0.043 × (WCFI × 100) − 0.037 × height + 0.015 × SBP − 0.010 × HR + 7.724*. In the RGT model, each 100-unit increase in the WCFI increased the RGT by 0.043 G (SE 0.015; 95% CI 0.009-0.078). The equation for estimating the SGT was as follows: *SGT = 0.103 × (WCFI × 100) − 0.069 × height + 0.018 × SBP + 15.899*. Thus, the SGT increased by 0.103 G for each 100-unit increase in the WCFI (SE 0.019; 95% CI 0.065-0.141). The findings indicated no significant differences between the observed and estimated value of the RGT (*P*=.49) or SGT (*P*=.80) when the predictive model was used (Table 7).

**Table 6:** Predictors of relaxed G tolerance (RGT) and straining G tolerance (SGT) in the multivariate linear regression.

Model and variables		*β*	SE	95% CI	*P* value
**RGT model**					
	Age	0.066	0.015	0.037 to 0.095	<.001
	WCFI^a^ × 100 (kg × G/bpm^b^)	0.043	0.017	0.009 to 0.078	.01
	Height (cm)	–0.037	0.009	–0.055 to –0.020	<.001
	SBP^c^ (mm Hg)	0.015	0.004	0.007 to 0.023	<.001
	HR^d^ (bpm)	–0.010	0.004	–0.017 to –0.001	.02
	Constant	7.724	1.516	4.736 to 10.712	<.001
**SGT model**					
	WCFI × 100 (kg × G/bpm)	0.103	0.019	0.065 to 0.141	<.001
	Height (cm)	–0.069	0.011	–0.091 to –0.048	<.001
	SBP (mm Hg)	0.018	0.005	0.008 to 0.028	<.001
	Constant	15.899	1.700	12.549 to 19.250	<.001

^a^WCFI: walking cardiac force index.

^b^bpm: beats per minute.

^c^SBP: systolic blood pressure.

^d^HR: heart rate.

**Table 7:** Comparison of the observed and estimated relaxed G tolerance (RGT) and straining G tolerance (SGT) values.

Variable	Estimated value, mean (SD)	Observed value, mean (SD)	*P* value
RGT	4.9 (0.4)	4.9 (0.9)	.49
SGT	7.9 (0.5)	7.9 (1.06)	.80

## Discussion

### Principal Findings

Several studies have measured HR responses to determine G tolerance [19-21]. We used the mHealth BioHarness device to collect HR data during physical activity performed before centrifuge training. Regarding the CFI values, the results revealed that the WCFI was positively related to G tolerance when the G level was increased at a gradual rate, which was consistent with other studies [12]. Additionally, this study successfully developed a new model for predicting G tolerance on the basis of changes in cardiac function. Age, height, resting blood pressure, and resting HR variables also influenced G tolerance.

### Age and Height

We observed that for every 1 extra year of age of the individuals undergoing centrifuge training, their RGT increased by 0.066 G. Older participants had higher G tolerance than younger participants, which was similar to the results of another study [22]. According to Webb et al [11], the RGT and SGT of fighter pilots in the US Air Force were both positively associated with age. In the Korean Air Force, older trainees were more likely to be able to tolerate 6 G exposure profile [8]. Several researchers have also observed that younger aircrew members, those with less flying experience, and those with fewer hours more frequently experience GLOC during flight [23-25].

Park et al [26] suggested that for well-experienced young aviators, age may not affect the frequency of GLOC episodes in centrifuge trials. In one study in the US Navy, Johanson et al [27] revealed that the mean age of those experiencing GLOC was not different from those not experiencing GLOC, which may be linked to past experience, aircraft type, flight maneuver, and situational awareness.

Older jet and fighter pilots often have more years of flying experience. Such pilots are also more frequently exposed to high-G forces during flight. Some evidence indicates that the cardiac performance of fighter pilots is higher after they have been repeatedly exposed to G force [28,29]. This adaptation to G force increases baroreflex activity and G tolerance by altering the G-time tolerance curve [30]. Therefore, our study participants may have had experience in adapting to G force in flight.

Because of greater hydrostatic pressure in taller people, height has been identified as a factor negatively affecting both G tolerance and sustained duration of G force exposure [10,11,31]. In a neutral standing posture, brain-level blood pressure is theoretically approximately 22 mm Hg lower than heart-level blood pressure in a 1 G environment. Thus, a longer distance between the brain and heart might mean lower blood pressure in taller aircrew. In agreement with previous findings, the height of our participants was negatively correlated with their G tolerance in our predictive model.

### SBP and HR

The heart ejects blood into cerebral tissue, and BP gradually decreases as blood travels further from the heart. Theoretically, elevated BP is conducive to modulating the effect of G stress. The cardiovascular system can sustain effective cerebral perfusion at up to approximately 4.5 to 5.5 G when the rate of increase is slow. However, the average resting SBP of our participants on the ground was approximately 140 mm Hg, which was slightly higher than usual. This may have been caused by the participants wearing the fitted AGS on their lower body and feeling stressed about their training. In our study, we also discovered that resting SBP was positively associated with RGT and SGT, similar to the US Air Force study that concluded that BP influences G tolerance [11].

In contrast to blood pressure, increased resting HR was disadvantageous for tolerating hypergravity. Our previous report similarly concluded that air force academy student pilots with elevated HR are less likely to tolerate a peak of 7.5 G when sustained for 15 seconds [9]. When arterial pressure and stroke volume drop due to exposure to high-G force, the sympathetic nerves trigger an increase in HR and strengthen cardiovascular function. Exertion levels during exercise can be determined using the maximum HR. The HR response is closely related to sport performance. By subtracting the participant’s age from 220, the target HR zones for different activities could be estimated. High-G training is a type of vigorous physical activity, and HR can rise to 160 bpm during G loading [7,9]. Nonetheless, if resting HR was elevated during the pretraining stage, the HR reserve (HRR) would be limited to a narrow range. HRR is one parameter of cardiovascular fitness. Consistent with some reports, we found that trainees with a lower HR or higher HRR were better able to resist the effects of G force [32,33]. This study verified the need to use mobile technology applications for obtaining cardiac data and understanding changes in the G tolerance levels of aviators.

### RGT and SGT

At slow acceleration, RGT is mainly determined by BP and baroreflexes. RGT typically ranges from 4.5 to 6 G and varies depending on the individual and the time [34]. When the G force surpasses the RGT, trainees initiate the AGSM to assist their cardiovascular system against the G stress. Inside the centrifuge, visual loss was subjectively assessed using a light bar. To avoid variation between participants, we used a large sample size. Our previous study indicated that the mean RGT and SGT were 5.1 and 7.8 G, respectively [12]. We identified nearly the same RGT and SGT values (RGT: 4.9 G and SGT: 7.9 G) in our sample of 213 participants.

We used the wearable mHealth BioHarness 3.0 device to record cardiovascular function and found that G tolerance was associated with the cardiac data. The CFI is composed of 3 factors, namely body weight, activity, and HR. Our findings indicated that cardiovascular responses on the ground can be used to predict the resistance of z-axis forces during exercise involving mild exertion. Research on the prevention of GLOC may focus on the development of a precaution system based on the CFI. Further monitoring of the CFI during G loading is recommended to track any instantaneous changes in the CFI prior to GLOC.

Until now, there is still no convenient and proper method to monitor the cardiac performance and G tolerance on the ground. Our study showed that the ability for G tolerance could be predicted by the WCFI. Because G tolerance changes every day, therefore, mobile technology combined with a wearable device is highly applicable to calculate the real-time WCFI and predict G tolerance. Military aircrew can directly understand their G tolerance anytime and anywhere by monitoring their cardiac health and performance via a mobile device during their daily activity. Before the flight, they can know their “low-G day” and maintain the good G awareness. Warning mechanisms based on the cardiac health recorded by a mobile device could be considered to develop and prevent in-flight GLOC and catastrophic mishap.

### Limitations

This study has some limitations. We included data obtained from women in our analysis, and our results suggest that gender did not have a significant effect on the outcome. However, this result may have been due to the small proportion of women. Second, for the calculation of the WCFI, the participants were asked to walk at their normal speed, but “normal” was subjective and their speed varied. Their HR values during walking were lower than 120 bpm, and the walking activity data covered a narrow range and exhibited a central tendency. Therefore, walking speed variation was unlikely to have significantly affected the outcomes. Although we have collected more data to develop and verify the predictive model, more participants are required to conduct an analysis and perform an external validation. Finally, depending on the airframes they were training on, aircrew had to have reached different levels and profiles relating to high-G training before they could participate in flight training. In this study, all participants met the standards of all the test profiles during training. Therefore, the authors could not clarify the relationship between the pass rate of high-G training and the CFI on the ground.

### Conclusions

Using mobile devices, we monitored the cardiac function of aircrew while they walked in a relaxed manner. We verified that the WCFI is positively associated with the level of G tolerance. Moreover, this study developed a model for estimating the G tolerance of military aircrew before they begin high-G training. The development and application of a WCFI-monitoring system for daily life could be considered to evaluate their G tolerance prior to flights.

## Abbreviations

AGS: anti-G suit
AGSM: anti-G straining maneuver
APRL: Aviation Physiology Research Laboratory
bpm: beats per minute
CFI: cardiac force index
CFR: cardiac force ratio
DBP: diastolic blood pressure
GLOC: G-induced loss of consciousness
HR: heart rate
HRR: heart rate reserve
mHealth: mobile health
RCFI: resting cardiac force index
RGT: relaxed G tolerance
SBP: systolic blood pressure
SGT: straining G tolerance
WCFI: walking cardiac force index
